# Forensic DNA databases in European countries: is size linked to performance?

**DOI:** 10.1186/2195-7819-9-12

**Published:** 2013-12-03

**Authors:** Filipe Santos, Helena Machado, Susana Silva

**Affiliations:** Research Centre for the Social Sciences, University of Minho, Minho, Portugal; Centro de Investigação em Ciências Sociais, Universidade do Minho, Instituto de Ciências Sociais, Campus de Gualtar, Braga, 4710-057 Portugal; Department of Sociology, University of Minho, Portugal; Centre for Social Studies, University of Coimbra, Coimbra, Portugal; Departamento de Sociologia, Instituto de Ciências Sociais, Campus de Gualtar, Braga, 4710-057 Portugal; Institute of Public Health of the University of Porto (ISPUP), Department of Clinical Epidemiology, Predictive Medicine and Public Health, University of Porto Medical School, Porto, Portugal; Departamento de Epidemiologia Clínica, Medicina Preditiva e Saúde Pública, Faculdade de Medicina, Al. Prof. Hernâni Monteiro, Porto, 4200-319 Portugal

**Keywords:** Forensic genetics, DNA database, Crime, Criminal law, Proportionality, Europe, Prüm decision

## Abstract

The political and financial investments in the implementation of forensic DNA databases and the ethical issues related to their use and expansion justify inquiries into their performance and general utility. The main function of a forensic DNA database is to produce matches between individuals and crime scene stains, which requires a constant input of individual profiles and crime scene stains. This is conditioned, among other factors, by the legislation, namely the criteria for inclusion of profiles and the periods of time and conditions for their retention and/or deletion. This article aims to provide an overview of the different legislative models for DNA databasing in Europe and ponder if wider inclusion criteria – and, consequently, database size – translates into more matches between profiles of crime scene stains and included individuals (performance ratio). The legislation governing forensic DNA databases in 22 countries in the European Union was analysed in order to propose a typology of two major groups of legislative criteria for inclusion/retention of profiles that can be classified as having either expansive effects or restrictive effects. We argue that expansive criteria for inclusion and retention of profiles do not necessarily translate into significant gains in output performance.

**Electronic supplementary material:**

The online version of this article (doi:10.1186/2195-7819-9-12) contains supplementary material, which is available to authorized users.

## Introduction

Forensic DNA databases constitute an important investigative resource in contemporary criminal justice systems. The centralised and computerised storage of DNA profiles in a database enables the systematic comparison and automated matching of crime scene samples and individual profiles. A forensic DNA database may help criminal investigators to establish links between a particular suspect of a specific crime and other unsolved crimes, or can provide support to identify potential suspects while clearing other suspects in the early stages of an investigation (Cutter 2006; Kaye 2006). In other words, forensic DNA databasing came to enable an active rather than reactive use of DNA technologies in criminal investigations (Williams and Johnson 2005). Thus, according to Chris Asplen (2004), forensic DNA databases allow for a “conservation of resources”, either by enabling crimes to be solved more quickly, or by speeding up the judicial proceedings by consolidating the evidence or introducing plea bargaining^1^.

There is also the argument that forensic DNA databases have the potential to prevent and deter crime (Cutter 2006), and this has been proclaimed in various jurisdictions as a justification for extending the inclusion criteria (Taylor et al. 2007). However, it has been noted by some authors that the potential deterrent effect and utility of a forensic DNA database has to take into account characteristics of criminal behaviour (e.g. Kazemian et al. 2010) and adaptations to forensic circumstances (e.g. (Beauregard and Bouchard 2010), and also that a DNA profile in a database may only be useful for a relatively short period of time – either because some criminal offenders in the database may have already ended their “criminal career”, or because those who have only just begun their criminal career may commit a variety of crimes for some time before their profile is identified and included in the database (Leary and Pease 2003; McCartney 2006).

The perceived value of forensic DNA analysis and databasing led the European Council to issue in February 1992 the non-binding Recommendation No. R (92) 1, in which it proposed a framework for the use of DNA in policies and legislation pertaining to European criminal justice systems (Council of Europe 1992). Most Member States have sought at least to produce legislation to regulate national forensic DNA databases, with rather distinct orientations on the establishment of criteria for inclusion and retention of profiles. At present, a considerable investment is being made in reinforcing international cooperation and exchanging genetic information in order to combat crime and terrorism, namely via the transposition into European law of the provisions contained in the Prüm Treaty (EU Council 2008a,2008b). While the necessary technological harmonisation is being achieved (Schneider 2009), differences in inclusion and retention criteria of the EU countries’ forensic DNA databases challenge standardisation regarding the retention of the profiles of children and innocent people, as well as the Treaty’s legitimacy and acceptability, or the lack of transparent disclosure of its operations (Prainsack and Toom 2013; McCartney et al. 2011). The construction of the Prüm regime can also generate power shifts in local practices, structures and actors, in what Prainsack and Toom (2010) have called dis/empowering effects. The authors exemplify by saying that while the Prüm regime can be empowering by actually reducing the circulation of personal information and providing benefits for crime victims, it can also prioritise the allocation of resources to certain crimes where biological traces are more likely to be found in detriment of others (Prainsack and Toom 2010).

Implementation and expansion of forensic DNA databases have been supported by success stories which emphasise the neutral and objective role of DNA technology in the resolution of crimes, while its limitations and ambiguities tend to go unnoticed in these narratives (Prainsack and Toom 2013). These stories tend to be grounded on two principles of what Robin Williams calls “forensic imaginary” – it is always possible to uniquely identify any object; and that any physical contact between two individuals implies transference of traces (Williams 2010: 135). Nevertheless, there is still insufficient evidence on the overall utility of forensic DNA databases, their impact in criminal deterrence or in securing convictions and, particularly, on the advantages of retaining profiles of people who were “at the wrong place, at the wrong time” (Cutter 2006; McCartney 2012; Human Genetics Commission 2009).

Two main types of studies have contributed to the analysis of the use of forensic science: studies that analyze the impact and effectiveness of physical evidence in criminal justice proceedings, and studies that focus on evaluating the performance of DNA databases.

There is a large body of empirical research on the use and relevance of physical evidence for the identification and prosecution of criminals, mostly developed in jurisdictions like England and Wales or the USA. This group of studies has recently been subjected to an extensive review by Ludwig and Fraser (2013) and, among published academic and institutional research about forensic science, also includes research studies that have focused on the effectiveness of DNA technologies in criminal investigations. The review of these studies indicates that it is important to “distinguish successes in individual cases from systematic benefits”, since most studies indicate that the effective use of forensic science is not consistent with general beliefs about its value (Ludwig and Fraser 2013: 6). This review also indicates the lack of systematic studies with robust experimental designs. As such, it illustrates the complexities and many caveats inherent to research on the effective use of forensic science and identifies limitations pertaining to methodological approaches, opportunistic sampling, demographical and geographical circumscription of the research, among others.

Wilson et al. (2010) have also focused on the effectiveness of DNA testing in criminal investigations by engaging in a systematic review of a selection of five field studies which included Roman et al.’s (Roman et al. 2008), Briody’s (2004), Schroeder’s (2007), Dunsmuir et al.’s (2008), and Tully’s (1998). While these studies provide empirical evidence of the relevance and value of DNA technologies for criminal investigation, they also expose the problematic aspects of engaging in an empirical study of this matter, like sample selection bias or generalizability of the results.

With increasing proportions of the population included in forensic DNA databases, several authors suggested that more research should be aimed at finding ways to evaluate and monitor their performance (Bieber 2006; Toom 2012; Walsh et al. 2008,2010; McCartney 2006; Leary and Pease 2003). In this second group of literature that sought to construct measures for assessing the performance of DNA databases there is the work of the forensic scientists Schneider and Martin (2001) who made a first assessment of European forensic DNA databases, stating that the value of forensic DNA databases, in terms of matches, was related to their number of records (Schneider and Martin 2001). However, there are multiple factors that can affect the performance of DNA databases and Frederick Bieber (2006) critically analysed several metrics and dimensions of utility to evaluate DNA databases. The author indicates that two metrics that have been used to measure the success of DNA databases, like “counting hits” and “investigations aided”, while interesting, have limited value in assessing the overall effectiveness, concluding that there should be more research into actual impacts on case outcomes, such as evaluating relevance for case resolution, the reduction of investigative costs or the exoneration of suspects (Bieber 2006).

Focusing on DNA database operation, Simon Walsh et al. (2008,2010) have sought to construct a model to assess DNA database performance and to evaluate and measure their output. These authors recognise that the evaluation and comparison of databases can be affected by several factors, like size and age of the database, as well as criminal behaviour and career span of the individuals whose profiles are included in the database. Moreover, the authors stress that “a limitation of any assessment of effectiveness is that only crude measures of return are available” (Walsh et al. 2008: 668). Nevertheless, Walsh et al.’s (2010) model suggests two critical parameters that are fundamental to optimise the performance of forensic DNA databases – the selection of included persons and crime scene stains.

Although acknowledging the limited significance of measuring the number of hits/matches obtained from a forensic DNA database for its wider impact in the criminal justice system, it can still be regarded as relevant, particularly when used for comparison among several jurisdictions. Additionally, the main factor involved in the operation of DNA databases is their legislative regulation, which is upstream from actual practices in the forensic evidentiary chain, which were not considered in this analysis. Thus, it is not the purpose of this article to measure the effectiveness of DNA databases, but rather to provide a wider consideration of the different criteria for inclusion and retention of profiles and their effects in terms of the relation between the included population and the number of matches produced. This approach is inherently limited because it does not contemplate actual inclusion and retention practices, only principles stated by law.

DNA databases in some jurisdictions retain the profiles of individuals who were never matched to a crime scene stain or, in some cases, were never formally accused of any punishable offense. Nevertheless, since legislation in some countries may allow for the inclusion in a database of DNA profiles of people who were never convicted or even formally accused as a suspect, these individuals can become symbolically and biologically categorised as a “risk” for the rest of the law-abiding population, and also searched as “suspects” for all future crimes (Simoncelli 2006). Bearing in mind a humanistic paradigm for offender rehabilitation, forensic DNA databases emerge as an important part of a biosurveillance apparatus which can be characterized, as Deleuze stated on his “Postscript on the Societies of Control” as a “limitless postponement” (Deleuze 1992). In other words, forensic DNA databasing enables a “morally accepted” inscription of suspicion (Lynch and McNally 2009), which can in some cases last indefinitely, insofar as an impersonal trace of the body is retained and previous suspects must “prove innocence” by not matching crime scene stains (Dahl and Sætnan 2009).

This article intends to contribute to the ongoing debate about forensic DNA databases, by considering European legislative dispositions that regulate these databases and analysing the person-stain match ratio in relation with the size of the database in European countries. First, we propose a typology based on an analysis of the conditions prescribed in the legislation that regulates the functioning of forensic DNA databases in 22 countries^2^ in the European Union (EU). This typology is drawn from the criteria for the inclusion of profiles and the periods of time and conditions for their retention and/or deletion. Second, by aggregating two major groups of countries where the legislation is classified as having either expansive or restrictive effects in terms of the prospective size of the forensic databases, we pondered if wider inclusion criteria renders a larger proportion of matches between profiles of crime scene stains and included individuals.

## Methods

Between October and December 2011, the authors collected information publicly available on the Internet for a comparative analysis of the legislation regulating the functioning of forensic DNA databases in the EU countries (listed in Additional file 1). In April 2013, the sources of the legislation were checked and no changes were found. The analysis of the legislation was carried out in terms of the criteria for the inclusion and removal of DNA profiles, namely: the type of crime committed; the maximum length of the potential sentence; the characteristics of the individual concerned and his/her likelihood of re-offending; and the periods and conditions for retention of profiles. Based on these criteria, the authors grouped together similar types of legislation, according to its expansive effects or restrictive effects in terms of the prospective size of the forensic DNA databases (Tables 1 and 2).

**Table 1:** **Criteria for the inclusion and removal of profiles in the restrictive group of countries**

Country	Criteria for inclusion of profiles	Criteria for removal of profiles
**Belgium**	Suspects and individuals convicted of serious crimes (list)	Convicted offenders – 30 years after inclusion; profiles in the "criminal investigation" database deleted when no longer needed
**France**	Suspects and individuals convicted of serious crimes (list)	Convicted offenders – 40 years after end of sentence or after individuals reach the age of 80; suspects – removed when retention is no longer considered necessary by a law official (or at the request of the party concerned)
**Germany**	Official suspects charged with crimes and individuals convicted of serious crimes or re-offending with other crimes	Profiles reviewed 10 years (adults), 5 years (young people) or 2 years (children) after inclusion. Removal of profiles of convicted offenders depends of a court decision
**Hungary**	Convicted offenders and individual suspected of crimes punishable with a sentence of > 5 years (or listed crimes involving lower sentences, such as drug trafficking)	Suspects – deleted after acquittal; convicted offenders – 20 after sentence has been served
**Ireland**	Suspects, convicted offenders (crimes punishable with a sentence of > 5 years or specific crimes involving lower sentences) and ex-convicts	Profiles of suspects acquitted or not charged removed after 10 years, or 5 years in the case of minors. Convicted offenders – indefinite retention
**Italy**	Individuals arrested, remanded in custody and convicted of premeditated crimes	Individuals arrested and remanded in custody – deleted on acquittal; convicted offenders – 20 years after the incident that led to sampling. No profile may be held for more than 40 years
**Luxemburg**	Individuals suspected of any crime (only by order of the court dealing with the case); convicted offenders – included only if sentenced for listed crimes or by order of the solicitor or court dealing with the case	Suspects –after acquittal, prescription of the crime or 10 years after death; convicted offenders – 10 years after death
**The Netherlands**	Suspects and individuals convicted of offenses or crimes for which preventative custody is allowed, or by a judicial order.	Convicted offenders – 30 years after sentencing if the crime is punishable with > 6 years or 20 after death; 20 years if < 6 years or 12 after death; Suspects and convicted of sexual offenses against minors – 80 years. Retention may be extended in the event of a new conviction; Suspects – DNA profiles are removed if they are not prosecuted or convicted (unless a match is found in the DNA database).
**Poland**	Suspects and convicted offenders (listed crimes)	Suspects – deleted after acquittal; convicted offenders – after 35 years
**Portugal**	Individuals convicted of premeditated crimes with an effective prison sentence of 3 years or more, by court order	Convicted offenders – until criminal record annulled
**Romania**	Suspects and convicted offenders (listed crimes)	Suspects – removed when retention no longer considered necessary by the courts or Public Prosecution; convicted offenders – retained until aged 60 (in the event of the death of the individual, retained for a further 5 years)
**Spain**	Individuals detained and those convicted of serious crimes (list)	Individuals detained – data deleted on prescription of crime^a^; individuals convicted – on date of prescription of criminal record (unless a court order states otherwise)
**Sweden**	Convicted offenders receiving non-financial sentences of over 2 years	Suspects – removed after acquittal; convicted offenders – 10 years after sentence served

^a^The period of prescription for the crime applies to individuals who are detained and for whom the judicial proceedings do not result in acquittal or conviction.

**Table 2:** **Criteria for the inclusion and removal of profiles in expansive group of countries**

Country	Criteria for inclusion of profiles	Criteria for removal of profiles
**Austria**	Individuals suspected and/or convicted of a dangerous assault^a^	Convicted: 5 years after death or at 80 years of age if the individual has not been forensically identified in the last 5 years; Minors: removed if s/he is not forensically identified in the previous three years; Acquitted suspects have to apply for removal and/or the authorities will decide if the acquitted suspect’s profile is no longer necessary.
**Denmark**	Suspects and individuals convicted of crimes punishable by sentences of > 1 year and 6 months	Convicted offenders – 2 years after death or at 80 years of age; suspects – 10 years after acquittal, at 70 years of age, 2 years after death
**Estonia**	Suspects and convicted offenders	Suspects and convicted offenders -10 years after death
**Finland**	Individuals suspected of crimes punishable with a sentence of > 6 months and offenders receiving sentences of > 3 years	Suspects – 1 year after acquittal (on the order of a legal officer) or 10 years after death; convicted offenders – 10 years after death
**Latvia**	Suspects and convicted offenders – any crime	Convicted offenders – 75 years; suspects – 10 years after verdict, if acquitted
**Lithuania**	Suspects and convicted offenders – any crime – and those temporarily detained	100 years after inclusion or 10 years after the death of the suspect or convicted offender
**Scotland**	Individuals detained for any crime	Suspects – deletion after acquittal or extension of retention period in cases of relevant sexual or violent offences; convicted offenders – indefinite retention
**Slovakia**	Suspects and convicted offenders – any crime	Convicted offenders – 100 years after the date of birth of the individual concerned; suspects – removal after acquittal
**United Kingdom (England, Wales)** ^**b**^	Individuals detained for any recordable offence	Indefinite retention

^a^In Austria, as defined in Section 16 (2) of the *Sicherheitspolizeigesetz* [Security Police Act], serious crimes are understood to be any threat against a legal asset by committing a premeditated crime punishable by law. In addition to the type of crime, the profile of an individual may be included when “the police cite the nature of the crime or the 'personality’ of the respective individual as grounds for expecting them to reoffend” (Prainsack and Kitzberger 2009).

^b^The *S. & Marper v. UK* decision of the ECHR is reflected on the *Protection of Freedoms Act 2012* in England and Wales, which will require all DNA samples to be destroyed within six months of being taken. It will still allow for speculative searches on the DNA profiles of non-convicted individuals, but eliminates their indefinite retention by establishing retention periods (until three years, plus two years if extended by court decision) differentiated according to the seriousness of the suspected offence. By October 2013, the Act will officially come into force.

Since all the eligible countries had repositories of legislation that could be accessed freely via the Internet, we consulted the original legislative texts in digital format. For the purposes of this article, the Czech Republic was excluded because it was not possible to find the relevant documentation^3^, and because the existing legislation (Police Act – Zákona o Policii [Law on Police] č. 283/1991) does not distinguish the dimensions required for comparison with the other countries. Estonia, Germany, Latvia, Lithuania and the Netherlands provide authorised English translations of the legislation, which facilitated reading and interpretation. In the eight cases in which documentation was only available in a language unfamiliar for the authors (Austria, Denmark, Finland, Hungary, Poland, Romania, Slovakia and Sweden), *Google Translate*™ (http://translate.google.com), an automatic translation application available on the Internet, was used. The serious limitations of publicly available data from the Internet were taken into consideration, and therefore the obtained information was systematically compared with other sources, namely reports produced by relevant agencies, such as Governmental bodies, professional and forensic experts associations, civil society groups and ethics councils (Scottish Government 2011; Bidasolo et al. 2009; Ministry of Justice 2007; Asplen 2012; Martin et al. 2001; Nuffield Council on Bioethics 2007; Council for Responsible Genetics 2011; Williams and Johnson 2005; Camp and Dierickx 2007).

Walsh et al. (2010) have devised an inferential model to identify and measure performance and efficiency parameters of forensic DNA databases. The authors found that the best independent measure of a database’s performance is obtained by dividing the number of hits by the product of the total individuals in the database and the number of crime scene samples. However, the ENFSI DNA Working Group (ENFSI 2012) argues that such measure wrongly suggests that larger databases are less efficient than smaller databases, because the result is always inversely proportional to the size of the database. Therefore, this group proposes two different performance parameters: the number of stain-to-person-matches relative to the number of persons included in the DNA database; and the number of stain-to-person-matches relative to the number of stains included in the DNA database (ENFSI 2012).

For the purpose of our analysis we chose to use the first parameter to compare the databases’ performance due to the limitations pertaining to the relevance of the stains in a database (Walsh et al. 2010), as well as the differences in actual deletion protocols for different countries (ENFSI 2012). Additionally, because the proposed typology focuses on the criteria that affects the number of included individuals and not on the quantity of crime scene stains.

Hence, the contents of Table 3 were adapted from a report produced by the *European Network of Forensic Science Institutes* (ENFSI) DNA Working Group (ENFSI 2012) and include a “performance ratio” (“person-stain matches per person” in the original) calculated from the most recent available data which dates from December 2011 (ENFSI 2011). This single parameter aims to indicate the performance of a DNA database by dividing the number of matches between individuals and crime scene stains by the total number of individuals included in the database (ENFSI 2012). Higher performance ratios can suggest that crime scene stains are being carefully selected and that the inclusion and retention criteria of the profiles of individuals are proportionately adequate. Thus, it can provide an indication whether the “right” people are being included in the database and/or irrelevant profiles are being deleted.

**Table 3:** **Population and DNA database performance**

Country	Total population	Total no. individuals included in DNA database	Proportion (%) of population included in database	Total no. crime scene stains	Person-stain matches	Date of data collection	Performance ratio
**RESTRICTIVE**							
**Belgium**	10.400.000	22.871	0.22%	26.237	1.886	Dec-11	**0.08**
**France**	64.300.000	1.873.016	2.91%	120.111	53.595	Dec-11	**0.03**
**Germany**	81.835.000	746.912	0.91%	201.955	99.974	Dec-11	**0.13**
**Hungary**	9.982.000	90.275	0.90%	2.264	240	Dec-11	**0.00**
**Luxemburg**	500.000	877	0.18%	662	200	Dec-11	**0.23**
**Netherlands**	16.100.000	130.067	0.81%	49.158	29.792	Dec-11	**0.23**
**Poland**	38.200.000	28.376	0.07%	2483	147	Dec-11	**0.01**
**Romania**	22.000.000	13906	0.06%	696	43	Dec-11	**0.00**
**Spain**	44.800.000	192.835	0.43%	59.761	20.671	Feb-12	**0.11**
**Sweden**	9.000.000	107.130	1.19%	23.539	32.144	Dec-11	**0.30**
**EXPANSIVE**							
**Austria**	8.100.000	151811	1.87%	48411	14.809	Dec-11	**0.10**
**Denmark**	5.500.000	77.500	1.41%	40.518	20.738	Sep-11	**0.27**
**Estonia**	1.400.000	29.274	2.09%	9.376	2.860	Jul-09	**0.10**
**Finland**	5.402.145	119.383	2.21%	13.296	14.779	Dec-11	**0.12**
**Latvia**	2.400.000	37037	1.54%	2092	761	Dec-11	**0.02**
**Lithuania**	3.169.000	55.561	1.75%	4.204	1.558	Dec-11	**0.03**
**Scotland**	5.062.000	236.202	4.67%	9.987	18.410	Jul-08	**0.08**
**Slovakia**	5.500.000	29.474	0.54%	6.686	3.454	Dec-11	**0.12**
**United Kingdom (England and Wales)**	53.700.000	5.508.170	10,26%	390.275	1.710.391	Dec-11	**0.31**

Note: The countries that did not provide sufficient data on the date when the information was collected by the ENFSI (Ireland, Italy and Portugal) have been excluded from this table.

Source: Adapted from ENFSI (2011).

## Results

Databases of all the analysed countries are used for criminal investigation purposes. In three of the countries – Belgium, Luxemburg and the Netherlands – the law specifically states that DNA analysis and comparison is made exclusively for the purposes of identification of persons who are, directly or indirectly, involved in a crime.

Considering the criteria for the inclusion and removal of DNA profiles from forensic databases stated on legislation of the 22 EU countries analysed in this article, it was possible to identify two main groups: countries with legislation classified as having expansive effects (Austria, Denmark, Estonia, Finland, Latvia, Lithuania, Scotland, Slovakia, and the United Kingdom (England and Wales)); and countries with legislation classified as having restrictive effects (Belgium, France, Germany, Hungary, Ireland, Italy, Luxemburg, the Netherlands, Poland, Portugal, Romania, Spain and Sweden). The characterisation of the two groups of legislation is described in Table 1 (restrictive countries) and Table 2 (expansive countries).

In the group of countries in which legislation is considered to have restrictive effects**,** the condition generally imposed for the inclusion of profiles in databases is that an individual is suspected or convicted of a crime that involves a potential or effective prison sentence, or the fact that the individual subjected to a collection of a biological sample has committed crimes that are considered serious (Table 1).

The criteria for inclusion and removal of profiles of the expansive countries are summarised in Table 2. This group is distinguished from the restrictive countries’ group for its comparatively lower thresholds for inclusion and lengthier retention periods for profiles, which may allow for a faster expansion in the number of profiles in the DNA database. In the expansive group, the inclusion criteria in most countries allow that individuals suspected of any crime can be submitted to sample collection and, consequently, to the inclusion of their respective DNA profiles in the forensic database. The exceptions are Denmark and Finland, where profile inclusion is associated with potential sentences, that is, the maximum prison sentence that may be given to an individual if convicted for a particular crime. The legislative configurations of the expansive countries appear to indicate the option increase the number of included individuals in the DNA database in order to enhance the number of person-stain matches. The basis for this option could be the argument that individuals who commit minor crimes or are identified as mere suspects may be involved in more serious crimes in the future. Moreover, that by creating legislative conditions for the expansion of a DNA database not only would improve the efficiency of criminal investigations but would also be likely to serve as a deterrent to crime (Nuffield Council on Bioethics 2007).

The expansive or restrictive effects of legislation can be observed from the higher or lower number of individuals (and/or the proportion of the total population) and crime scene stains included in the databases, respectively (Table 3). Differences in the median performance ratio were compared by the Mann–Whitney test, and they were not statistically significant (restrictive vs expansive: 0.095 vs. 0.100, p = 0.567).

**Figure 1:**
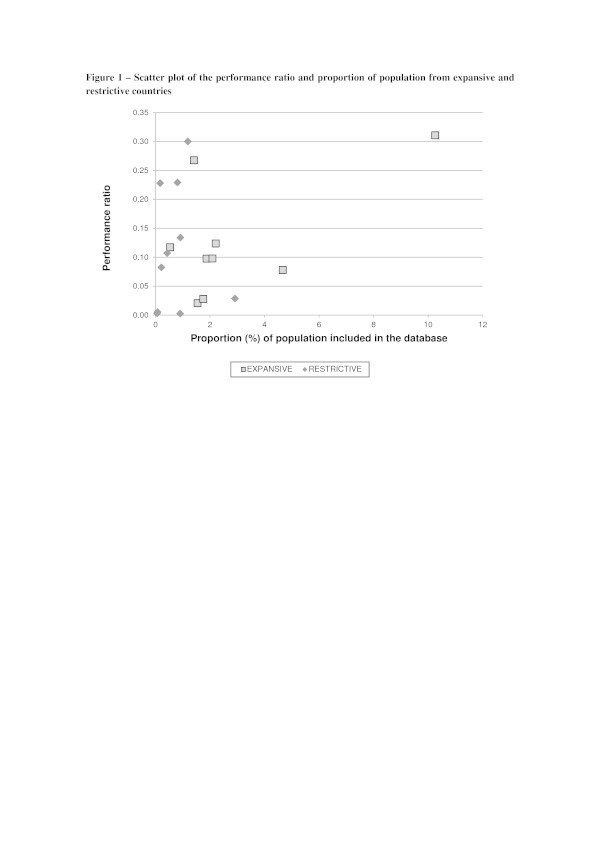
**Scatter plot of the performance ratio and proportion of population from expansive and restrictive countries.**

Available data do not support the idea according to which the size or the proportion of the total population that is included in a forensic DNA database is linked to its output in terms of measured person-stain matches (Figure 1).

## Discussion

The comparative analysis of the legislation that regulates the functioning of forensic DNA databases in 22 countries in the European Union emphasised a distinction between what we have argued to be expansive and restrictive effects, since the proportion of the population included in the expansive group is generally larger than in the restrictive group (Table 3). Previous studies have sought to assess and measure the effectiveness of the contribution of forensic science to criminal justice and the efficiency of DNA databases. As discussed in the introduction, there is a lack of systematic empirical research studies, and studies made to date stress the conceptual and methodological complexity of assessing the actual impacts of forensic science and DNA technology in justice systems. Moreover, most research has been centred on jurisdictions like England and Wales, the USA and Australia. This aim of this article is not to provide a different method to measure or assess the impact of DNA databases, but rather to analyse European countries’ legislative criteria for inclusion and retention of profiles and then to consider the effects in terms of the association between the number of individuals included and the number of person-stain matches.

By comparing the performance ratio with the proportion of the population included (Figure 1) the data show that a DNA database’s performance in terms of person-stain matches is not linked to its size in terms of individuals included. The countries with the highest proportion of their population included in the database do not necessarily have a significantly higher “performance ratio” than the countries with comparatively lower proportion of included individuals. In other words, a high “performance ratio” of person-stain matches per person indicates that the “right” people are being included in the database (ENFSI 2012), meaning that the included individuals are the ones that present a good chance of matching crime scene stains.

There are limits to this comparison, since it does not account for local police and forensic practices and contingencies in the application of the legislation, and because it does not take into account the date of implementation of the databases, methods of match counting, crime rates or policy changes over time.

Nevertheless, the proposed classification of expansive and restrictive effects of the legislation is still valid insofar it emphasizes the underlying principles and envisioned purposes of the diverse European forensic DNA databases. Furthermore, by considering the proportion of population included and the number of person-stain matches per person, it can also be useful to ponder if there is a relevant association between them, insofar it may raise the question of the proportionality of the inclusion and retention criteria prescribed in the DNA database legislation of the “expansive” countries (Nuffield Council on Bioethics 2007). This is particularly relevant since automated DNA searches and exchange of information between EU countries was enacted into EU law through the so-called Prüm decisions. As such, a virtual EU forensic DNA database constructed with different legislative understandings of proportionality, bodily integrity, right to individual privacy and presumption of innocence, may present an example of increasing inequalities among EU’s “biological citizens” (Plows and Boddington 2006; Prainsack and Toom 2013). The *S. & Marper v. UK* decision of the ECHR, which binds the signatory countries to its ruling, is thus far the most important decision regarding DNA databases in Europe. It argues against the NDNAD’s blanket and indiscriminate retention of DNA samples and profiles of non-convicted individuals under the justification of the database’s expansion in order to enhance public protection. In the ECHR’s view, the indefinite retention of non-convicted person’s data is stigmatizing and interferes with the individual right to privacy. Thus, the *S. & Marper v. UK* decision of the ECHR demands that the contracting states must find a fair balance between the competing public and private interests.

While, intuitively, one would agree with Schneider and Martin’s (2001) statement that the value of a forensic DNA database is associated to its number of records, it would appear that at least one dimension of utility – identifying offenders from stains collected at crime scenes – might depend more on the characteristics of the individuals that are registered rather than on their number, which is largely conditioned by the existing governing legislation.

The success of a considerable number of person-stain matches in the first operational years of databases can be compared to the initial success of the exchange of information between some members of the Prüm Treaty when many unidentified crime scene stains were identified, allowing cross-border crimes to be solved (McCartney et al. 2011). Similarly, the establishment of a forensic DNA database can provide useful intelligence for solving earlier crimes once crime scene stains are loaded and compared to the profiles of included individuals. For example, an old stain collected at the scene of an unsolved case of a robbery can be matched to the profile of an individual who is presently being charged or convicted for other crimes.

Therefore, we conclude that while, theoretically, the effort to maximise the number of included individuals in a forensic DNA database would appear to be an effective strategy to enhance its value and utility, the comparison of person-stain match figures in the context of the typology proposed in this article would indicate that the expansion of a forensic DNA database does not necessarily translate into greater output performance in terms of person-stain matches. Hence, it is our understanding that there is a need for further studies on the actual criminological and judicial impacts of forensic DNA profiling and databasing.

## Endnotes

^1^Plea bargaining tends to be associated with adversarial legal systems and refers to negotiations involving an admission of guilt by the accused in return for more favourable treatment by the courts. This process has been criticised on the grounds that it negates the principle of presumption of innocence and increases the risk of an innocent person being convicted, particularly when they do not believe they have much chance of being acquitted due to the fact that they are economically disadvantaged and/or a member of an ethnic minority group (Siegel 2010).

^2^Scotland has its own legislation and a DNA database that is separate from the United Kingdom (England and Wales) database. For the purposes of this paper, information on Scotland is therefore treated as belonging to a separate country and is considered separately from information on the United Kingdom.

^3^Since the original legislation could not be found, searches were made using other sources. However, it was not possible to find the Police President Binding Instruction No. 88/2004 referred to in Camp and Dierickx (Camp and Dierickx 2007).

## Electronic supplementary material

Additional file 1: **Appendix List of legislative sources.** (PDF 68 KB)
