# Supplementary material for: Forensic DNA databases in European countries: is size linked to performance?
Source: Life Sci Soc Policy. 2013 Dec 3;9:12. doi: 10.1186/2195-7819-9-12 (PMC4513018; doi:10.1186/2195-7819-9-12)
Supplement: Supplementary file 1 — Additional file 1: Appendix List of legislative sources. (PDF 68 KB) [file 40504_2013_12_MOESM1_ESM.pdf]

Appendix – List of legislative sources

Austria - Sicherheitspolizeigesetz

Belgium - Loi Relative à la Procédure d'Identification par Analyse ADN en Matière Pénale

Denmark – 434/2000 - Lov om oprettelse af et centralt dna-profilregister

Estonia - Riikliku DNA registri asutamine ja registri pidamise põhimäärus

Finland - Pakkokeinolaki (Coercive Measures Act 450/1987)

France - Code de Procédure Pénale (Code of Criminal Procedure - ENG)

Germany - German Code of Criminal Procedure (ENG)

Hungary – 2009. évi XLVII. törvény (Law on Criminal Records)

Ireland - Criminal Justice (Forensic Evidence and DNA Database System) Bill 2010 as Initiated and Explanatory Memorandum

Italy - Istituzione della Banca Dati Nazionale del DNA e del Laboratorio Centrale per la Banca Dati Nazionale del DNA

Latvia - Law on Development and Use of the National DNA Database (ENG)

Lithuania - 2011 m. sausio 20 d. Nr. 5-V-42 (Order N.º5-V-42 Establishing provisions for DNA data and records)

Luxembourg - Loi du 25 Août 2006 Relative Aux Procédures d'Identification par Empreintes Génétiques en Matière Pénale et Portant Modification du Code d'Instruction Criminelle

The Netherlands - Wet DNA-Onderzoek Bij Veroordeelden; The Dutch DNA Testing (Convicted Persons) Act (ENG)

Poland – Kodeks postępowania karnego (Law on the Police – Code of Criminal Procedure)

Portugal – Lei 5/2008 – Aprova a criação de uma base de dados de perfis de AND para fins de identificação civil e criminal

Romania – Lege 76/2008 - privind organizarea și funcționarea Sistemului Național de Date Genetice Judiciare

Scotland - Criminal Justice and Licensing (Scotland) Act 2010; Criminal Procedure (Scotland) Act 1995

Slovakia – Law 417/2002 on the use of analysis of deoxyribonucleic acid to identify people

Spain - Ley Orgánica 10/2007, de 8 de Octubre, Reguladora de la Base de Datos Policial Sobre Identificadores Obtenidos a Partir del ADN

Sweden – Polisdatalag (2010:361)

United Kingdom (England and Wales) – Police and Criminal Evidence Act 1984
